# Medroxyprogesterone acetate inhibits interleukin 6 secretion from KPL-4 human breast cancer cells both in vitro and in vivo: a possible mechanism of the anticachectic effect

**DOI:** 10.1038/sj.bjc.6690099

**Published:** 1999-02

**Authors:** J Kurebayashi, S Yamamoto, T Otsuki, H Sonoo

**Affiliations:** Department of Breast and Thyroid Surgery, Kawasaki Medical School, Kurashiki, Okayama, 701-0192, Japan; Department of Hygiene, Kawasaki Medical School, Kurashiki, Okayama, 701-0192, Japan

**Keywords:** interleukin 6, cachexia, medroxyprogesterone acetate, breast cancer, cell line

## Abstract

Interleukin 6 (IL-6) is a multifunctional cytokine. Recent reports suggest that circulating IL-6 secreted from tumour cells plays an important role in cancer-induced cachexia. Medroxyprogesterone acetate (MPA) has been used as an endocrine therapeutic agent for patients with breast cancer. It has been suggested that MPA decreases serum IL-6 levels and preserves the bodyweight of patients with advanced breast cancer. However, the mechanisms of action responsible for the anticachectic effect of MPA have not been elucidated. Therefore, the effects of MPA on IL-6 secretion were studied both in vitro and in vivo using a human breast cancer cell line, KPL-4, which secretes IL-6 into medium and induces cachexia when injected into female nude mice. MPA (10–1000 nM) dose-dependently decreased basal IL-6 secretion into medium, and also suppressed tumour necrosis factor (TNF-α)-induced IL-6 secretion. Both basal and TNF-α-induced IL-6 mRNA levels were dose-dependently lowered by MPA. Moreover, intramuscular injections of MPA (100 mg kg^−1^ twice a week) into nude mice bearing KPL-4 transplanted tumours significantly decreased serum IL-6 levels without affecting tumour growth and preserved the bodyweight of recipient mice. These findings suggest that suppression of IL-6 secretion from tumour cells, at least in part, causes the anticachectic effect of MPA. © 1999 Cancer Research Campaign


*British Journal of Cancer* (1999) **79**(3/4), 631–636
© 1999 Cancer Research Campaign
Article no. bjoc.1998.0099

### Medroxyprogesterone acetate inhibits interleukin 6 secretion from KPL-4 human breast cancer cells both in vitro and in vivo: a possible mechanism of the anticachectic effect

**J Kurebayashi[1], S Yamamoto[1], T Otsuki[2] and H Sonoo[1]**

[1]Department of Breast and Thyroid Surgery and [2]Department of Hygiene, Kawasaki Medical School, Kurashiki, Okayama 701-0192, Japan

**Summary** Interleukin 6 (IL-6) is a multifunctional cytokine. Recent reports suggest that circulating IL-6 secreted from tumour cells plays an important role in cancer-induced cachexia. Medroxyprogesterone acetate (MPA) has been used as an endocrine therapeutic agent for patients with breast cancer. It has been suggested that MPA decreases serum IL-6 levels and preserves the bodyweight of patients with advanced breast cancer. However, the mechanisms of action responsible for the anticachectic effect of MPA have not been elucidated. Therefore, the effects of MPA on IL-6 secretion were studied both in vitro and in vivo using a human breast cancer cell line, KPL-4, which secretes IL-6 into medium and induces cachexia when injected into female nude mice. MPA (10–1000 nM) dose-dependently decreased basal IL-6 secretion into medium, and also suppressed tumour necrosis factor (TNF-α)-induced IL-6 secretion. Both basal and TNF-α-induced IL-6 mRNA levels were dose-dependently lowered by MPA. Moreover, intramuscular injections of MPA (100 mg kg$^{-1}$ twice a week) into nude mice bearing KPL-4 transplanted tumours significantly decreased serum IL-6 levels without affecting tumour growth and preserved the bodyweight of recipient mice. These findings suggest that suppression of IL-6 secretion from tumour cells, at least in part, causes the anticachectic effect of MPA.

**Keywords:** interleukin 6; cachexia; medroxyprogesterone acetate; breast cancer; cell line

Cancer-induced cachexia is an uncomfortable paraneoplastic syndrome which worsens the quality of life of patients with advanced malignancies. A number of studies have been conducted to explore the factors responsible for this cachexia, and it has been revealed that elevated blood levels of tumour necrosis factor $\alpha$ (TNF-$\alpha$), interleukin 1 (Il-1), IL-6, IL-11, $\gamma$-interferon, leukaemia inhibitory factor or 24 kDa proteoglycan may induce cachexia (Beutler et al, 1986; Moldawer et al, 1988; Metcalf et al, 1990; Matthys et al, 1991; Strassmann et al, 1992*a*; Ohsumi et al, 1994; Todorov et al, 1996). One of these factors, IL-6, is a pleiotropic cytokine physiologically involved in the differentiation of myeloid and neuronal cells, and pathologically involved in the early host response to infection and injury (Kishimoto, 1989), in the proliferation of myeloma (Kawano et al, 1988), urological cancer cells (Miki et al, 1989; Okamoto et al, 1997*a,b*) and possibly breast cancer cells (Chiu et al, 1996), and in the development of hypercalcaemia and osteolytic metastases (De La Mata et al, 1995). A series of studies have suggested that IL-6 secreted from tumour cells is one of the cachectic factors in animal models of murine colon cancer or of xenograft tumours in athymic nude mice (Greenberg et al, 1992; Strassmann et al, 1992*b*; Ohe et al, 1993; Fujimoto-Ouchi et al, 1995; Yasumoto et al, 1995; Billingsley et al, 1996; Kajimura et al, 1996; Mori et al, 1996; Ohira et al, 1996; Tsujinaka et al, 1996). Recently, clinical reports have also suggested that elevated IL-6 levels in the sera of patients with oesophageal cancer correlate with their bodyweight loss (Oka et al, 1996), and that a decrease in serum IL-6 levels induced by medroxyprogesterone acetate (MPA) correlates with a reversion of bodyweight loss in patients with advanced breast cancer (Yamashita et al, 1996). The former report noted that IL-6 is overexpressed in tumour cells and suggested that IL-6 secreted from the tumour cells may be one of the causes of cancer-induced cachexia. However, the latter report did not explore the mechanisms responsible for the reduction in the serum IL-6 levels of patients induced by MPA.

MPA has been used as an endocrine therapeutic agent for the treatment of hormone-responsive breast cancer. One of the adverse effects of MPA is bodyweight gain of the patients (Van Veelen et al, 1986). It has been reported, however, that concomitant administration of MPA reduces the nausea and anorexia induced by the administration of cytotoxic agents, and subsequently reverses bodyweight loss (Tominaga et al, 1994). Based on these findings, a clinical trial using MPA as an anticachectic agent has been undertaken in patients with various malignancies which are not hormone responsive (Downer et al, 1993).

To elucidate the mechanisms of action of the anticachectic effect of MPA, its effects on IL-6 secretion from the KPL-4 human breast cancer cell line were explored both in vitro and in vivo in this study. This KPL-4 cell line was recently established in our laboratory and is the first human breast cancer cell line to secrete IL-6 into medium and to induce cachexia when injected into female athymic nude mice (Kurebayashi et al, 1997). The experimental results in this study suggest that an inhibitory effect of MPA on IL-6 secretion from tumour cells may, at least in part, cause the anticachectic effect of MPA.

*Received 2 March 1998*
*Revised 27 May 1998*
*Accepted 28 May 1998*

*Correspondence to:* J Kurebayashi, Department of Breast and Thyroid Surgery, Kawasaki Medical School, 577 Matsushima, Kurashiki, Okayama 701-0192, Japan

### MATERIALS AND METHODS

### Cell line and cell culture

The KPL-4 cell line was recently established and its characterization has been published elsewhere (Kurebayashi et al, 1997). In brief, this cell line was derived from the malignant pleural effusion of a Japanese patient with recurrent breast cancer. The recurrent disease exhibited an inflammatory skin metastasis. All the Erb B family receptors are expressed in this cell line. Neither oestrogen nor progesterone receptors are expressed. Injections of this cell line into the mammary fat pad of female nude mice produce rapid-growing tumours and induce severe cachexia. Immunoreactive IL-6 is detected in both culture medium and the serum of recipient mice. KPL-4 cells are routinely cultured in Dulbecco's modified Eagle medium (DMEM) supplemented with 5% fetal bovine serum (FBS). The population-doubling time is approximately 48 h in the routine medium. For IL-6 measurement of the culture medium, $1 \times 10^5$ KPL-4 cells per well were inoculated into 12-well plates (SB Medical, Tokyo, Japan) with the routine medium. The medium was changed to DMEM supplemented with 5% FBS (ICN Biochemicals, Costa Mesa, CA, USA) plus 10–1000 nM MPA (kindly provided by Kyowa Hakko, Tokyo, Japan) or 0.1–10 ng $ml^{-1}$ TNF-$\alpha$ (Gibco, Gaithersburg, MD, USA), or both, 4 days after the inoculations. The culture medium and cells were collected after a 48-h incubation. The IL-6 concentration in the medium was measured as described below. After cell dispersion with 0.05% trypsin plus 0.02% EDTA in phosphate-buffered saline (PBS) for over 10 min, the cell number per well was measured with a Coulter counter (Coulter Electronics, Harpenden, UK). For RNA extraction, semiconfluent KPL-4 cells in T-25 flasks (Corning, Tokyo, Japan) were incubated with DMEM supplemented with 5% FBS plus 100–1000 nM MPA, 10 ng $ml^{-1}$ TNF-$\alpha$ or both for 48 h, and then the treated cells were collected with a trypsin/EDTA solution. After washing with PBS, the cell pellet was stored at –80°C before use.

### IL-6 measurement

IL-6 concentrations in cultured medium and mouse serum were measured with a chemiluminescent enzyme immunoassay kit (Fujirebio, Tokyo, Japan) according to the manufacturer's recommendations. Briefly, a mouse anti-human IL-6 monoclonal antibody (HH 61-10) was used as the first antibody, and a mouse anti-human IL-6 monoclonal antibody labelled with alkaline phosphatase (HH 61-2 Fab′) was used as the second one. After removing the unbound second antibody, 3-(2′-spiroadamantane)-4-methoxy-4-(3″-phosphoryloxy)phenyl-1,2-dioxetane disodium salt was added. Chemiluminescence was measured with a Lumipulse luminometer (Fujirebio). As the standard, 20–1000 pg $ml^{-1}$ of human recombinant IL-6 was used. In this assay, no cross-reactivity against recombinant mouse IL-6 was observed. The minimal detectable concentration of IL-6 was 0.2 pg $ml^{-1}$. The intra-assay coefficients of variation for the high, middle and low sample levels were 2.8%, 2.2% and 3.8% respectively. The interassay coefficients of variation for these sample levels were 3.6%, 4.9% and 8.6% (Takemura et al, 1996). Because the IL-6 concentration in the fresh medium was undetectable and increased linearly for at least 2 days, IL-6 secretion into the medium was defined as follows:

$$\text{IL-6 secretion per cell per 48 h} = \frac{\text{Concentration of IL-6} \times \text{volume of medium}}{\text{Mean cell number}}$$

### Semiquantitative reverse transcription polymerase chain reaction (RT-PCR) for IL-6 mRNA

Total cellular RNA from KPL-4 cells was extracted with a TRIzol RNA extraction kit (Gibco) according to the manufacturer's recommendations. One microgram of total RNA and oligo (dT)18 primer (final concentration 1 $\mu$M) in 12.5 $\mu$l of diethyl pyrocarbonate-treated water was heated to 70°C for 2 min followed by cooling on ice for 1 min. cDNA synthesis was initiated with 200 units of recombinant Molony-murine leukaemia virus reverse transcriptase (Clontech Laboratories, Palo Alto, CA, USA) under conditions recommended by the manufacturer, and the reaction was allowed to proceed at 42°C for 1 h. The reaction was terminated by heating at 94°C for 5 min. cDNA was finally dissolved to a final volume of 100 $\mu$l by adding 80 $\mu$l of diethyl pyrocarbonate-treated water and was frozen at –20°C before use. Oligonucleotide primers for RT-PCR were designed using published sequences of human IL-6 and $\beta$-actin, and were synthesized by the solid-phase triester method. The primers used in this study were: IL-6, 5′-GAACTCCTTCTCCACAAGCG-3′ (sense) and 5′-GAATCCAGATTGGAAGCATCC-3′ (antisense); and $\beta$-actin, 5′-TGACGGGGTCACCCACACTGTGCCCATCTA-3′ (sense) and 5′-CTAGAAGCATTTGCGGTGGACGATG-GAGGG-3′ (antisense). The expected sizes from reported cDNA sequences were 316 bp and 610 bp for the IL-6 and $\beta$-actin genes respectively. Each RT-PCR reaction contained 1:100 of cDNA (equivalent to a cDNA amount from 10 ng of initial total RNA), 200 nM concentrations of each primer, 200 $\mu$M deoxynucleotide triphosphates, 10 mM tris-HCl (pH 8.8), 1.5 mM, magnesium chloride, 50 mM potassium chloride, 0.08% Nonident P40, and one unit of recombinant Thermus aquatics DNA polymerase (MBI Fermentas, Vilnius, Lithuania) in a final volume of 20 $\mu$l. After an initial denaturation at 94°C for 4 min, 25 cycles of denaturation for $\beta$-actin and 35 cycles for IL-6 (94°C for 15 s), annealing (60°C for $\beta$-actin and 58°C for IL-6 for 15 s), and extension (72°C for 30 s) were performed on a DNA Thermal Cycler 2400 (PC-960G Microplate Gradient Thermal Cycler, Mortlake, Australia). The final extension was performed for 5 min. The number of cycles in the RT-PCR was determined to obtain logarithmic amplification of both genes for semiquantitative analysis of the expression levels of the genes. After visualization of the RT-PCR products by 1.2% agarose gel stained with ethidium bromide, gel images were obtained using the FAS-II UV-image analyzer (Toyobo, Tokyo, Japan), and the densities of the products were quantified using the Quantity One version 2.5 (PDI, Huntington Station, NY, USA). The relative expression levels were calculated as the density of the product of the IL-6 gene divided by that of the $\beta$-actin gene from the same cDNA.

### Animal experiments

Semiconfluent KPL-4 cells were trypsinized and harvested, and viable cells were counted in a haemocytometer using trypan blue exclusion. To investigate the effects of MPA on KPL-4 tumour growth, IL-6 secretion and mouse cachexia, approximately $2 \times 10^6$ KPL-4 cells were injected into the right and left mammary fat pads of 4-week-old female nude mice (Clea, Tokyo, Japan). In the MPA-treated group, 100 mg $kg^{-1}$ MPA was intramuscularly administered twice every week for between 3 and 7 weeks after the cell injections. The same volumes of vehicle (sesame oil) were administered in the control group in the same manner. Five mice (ten tumours) were treated in each group. Three-dimensional tumour size was measured with calipers every week after the cell

© Cancer Research Campaign 1999

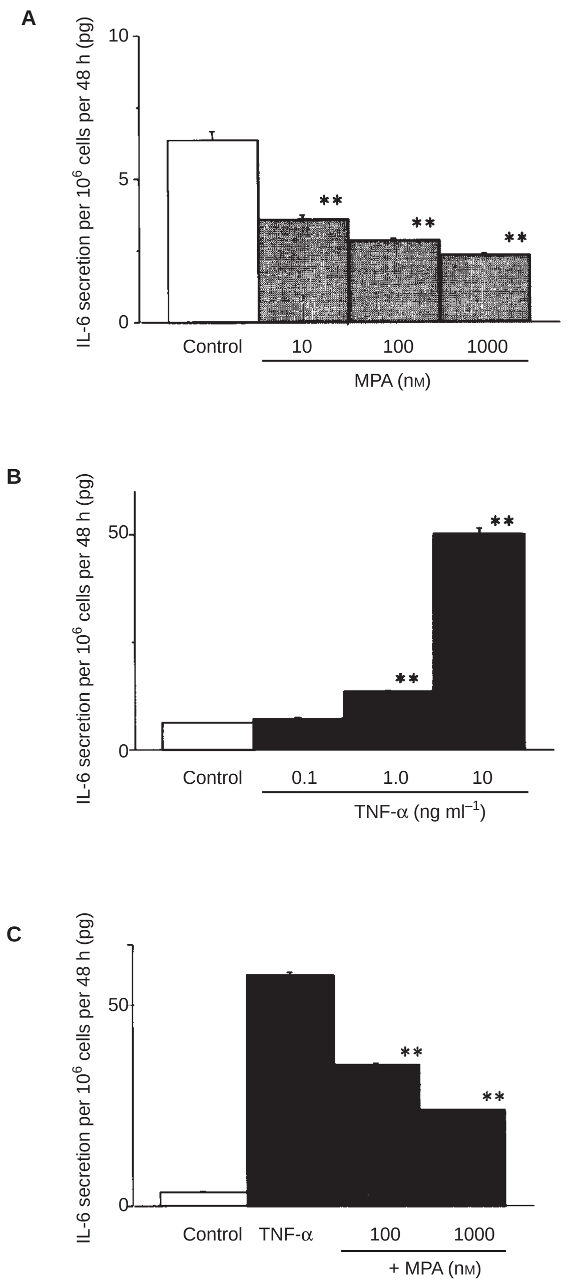

**Figure 1** Effect of MPA (**A**), TNF-$\alpha$ (**B**) and their combination (**C**) on IL-6 secretion into medium from KPL-4 human breast cancer cells. Semiconfluent cells were incubated with the routine medium with 10–1000 nM MPA, 0.1–10 ng ml$^{-1}$ TNF-$\alpha$, or 10 ng ml$^{-1}$ TNF-$\alpha$ plus 100–1000 nM MPA for 48 h. IL-6 concentrations in the medium and cell number per well were measured, and IL-6 secretion per cells per 48 h was calculated as described in Materials and methods. The values represent means ± s.e. of triplicate samples. $^{**}P < 0.01$ compared with respective controls (**A, B**) or the TNF-α-stimulated value (**C**)

injections. The bodyweight of mice was measured every week before the administration. Tumour volume was calculated as the product of the largest diameter, the orthogonal measurement and the tumour depth. Mean tumour volume was calculated as the sum of tumour volumes divided by the number of tumours. All mice were sacrificed by cervical dislocation 7 weeks after the cell injections. After measurement of tumour weight, tumour samples were fixed with 5% buffered formalin and embedded in paraffin for morphological analysis. Then, mouse blood was collected, and the serum was stored at –80°C before use.

To clarify the induction of mouse cachexia by transplantation with KPL-4 cells, the bodyweight of intact female mice was measured every week and the recovery of bodyweight loss after tumour removal was observed in separate experiments.

The animal protocols for these experiments were approved by the Animal Care and Use Committee of Kawasaki Medical School.

### Statistical analysis

The IL-6 secretion, tumour volume, tumour weight, mouse body weight and serum IL-6 concentration of mice were expressed as means ± s.e. These values for the control and treated groups were compared using ANOVA with StatView computer software (ATMS, Tokyo, Japan). All the experiments in this study were repeated at least twice and the reproducibility of the results was confirmed.

### RESULTS

### MPA reduces both IL-6 secretion and IL-6 mRNA level in KPL-4 cells

As shown in Figure 1A, a 48-h incubation with 10–1000 nM MPA dose-dependently decreased basal IL-6 secretion into medium from KPL-4 cells to 0.57-fold for 10 nM, 0.45-fold for 100 nM, and 0.37-fold for 1000 nM. In contrast, TNF-$\alpha$ dose-dependently stimulated IL-6 secretion to 1.17-fold for 0.1 ng ml$^{-1}$, 2.17-fold for 1.0 ng ml$^{-1}$, and 8.07-fold for 10 ng ml$^{-1}$ (Figure 1B). TNF-α-stimulated IL-6 secretion was dose-dependently inhibited by MPA to 0.61-fold for 100 nM MPA and 0.42-fold for 1000 nM MPA (Figure 1C). In addition, changes in IL-6 mRNA levels were investigated by a semiquantitative RT-PCR method. As shown in Figure 2A, 48 h incubation with 100 or 1000 nM MPA dose-dependently lowered the basal IL-6 mRNA expression level in KPL-4 cells. The relative IL-6 expression ratios compared with $\beta$-actin expression (internal control) were 0.95 for the control, 0.20 for 100 nM MPA and 0 for 1000 nM MPA. The IL-6 mRNA level induced by TNF-$\alpha$ was also inhibited by MPA (Figure 2B).

### MPA decreases serum IL-6 levels and preserves bodyweight of female nude mice bearing KPL-4 tumours

Intramuscular injections of 100 mg kg$^{-1}$ MPA twice a week for 4 weeks did not significantly affect KPL-4 tumour growth, but did significantly preserve mouse body weight (Figure 3A and B). Tumour weights and bodyweights 7 weeks after the cell injections in each group were 0.43 ± 0.11 g and 19.2 ± 0.7 g for the control group and 0.47 ± 0.08 g and 23.2 ± 0.7 g for the MPA-treated group respectively ($P = 0.013$ comparing the two groups for bodyweight).

The bodyweight in the control group was significantly lower than that of the intact mice and that of the MPA-treated group (Figure 3B). No difference in bodyweight was observed between the intact mice and MPA-treated mice. In addition, the mice bearing KPL-4 transplanted tumours completely recovered bodyweight loss after tumour removal (data not shown). These findings suggest that the mouse bodyweight loss was induced by the KPL-4 transplanted tumours, and that MPA-treatment resulted in

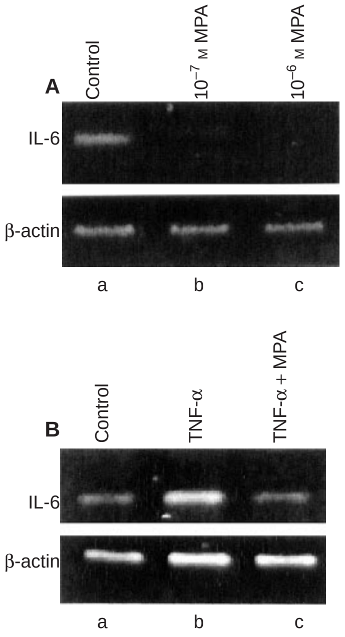

**Figure 2** Semiquantitative RT-PCR for the expression of IL-6 mRNA. One microgram of total RNA extracted from untreated or treated KPL-4 cells was reverse transcribed, and 1:100 of cDNA was amplified by PCR. Primers specific for IL-6 and $\beta$-actin (internal control) cDNAs generated fragments of 316 bp and 610 bp respectively. (**A**) a, control; b, treated with 100 nM MPA; c, treated with 1000 nM MPA. (**B**) a, control; b, treated with 10 ng ml$^{-1}$ TNF-$\alpha$; c, treated with 10 ng ml$^{-1}$ TNF-$\alpha$ plus 1000 nM MPA

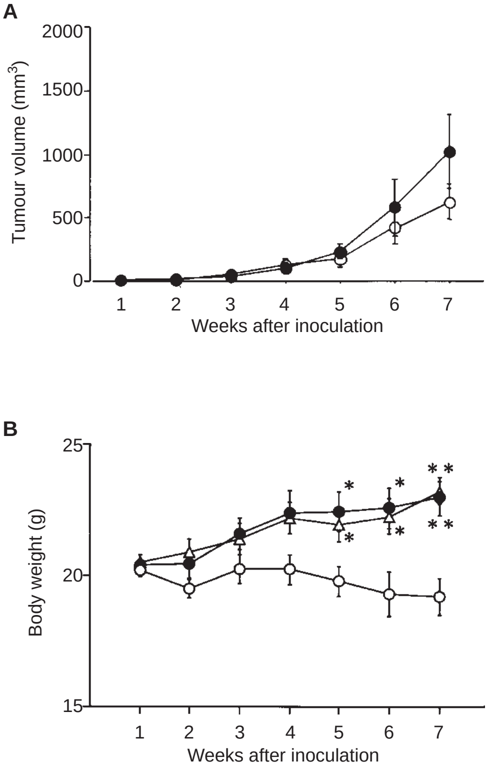

**Figure 3** Effect of MPA on tumour growth (**A**) and bodyweight (**B**) in nude mice bearing KPL-4 transplanted tumours. Approximately $2 \times 10^6$ cells were injected into the mammary fat pad of 4-week-old athymic nude mice. MPA (100 mg kg$^{-1}$) was administered i.m. twice a week from 3 to 7 weeks after the cell inoculations. Bodyweight of intact mice was also measured to clarify the cachectic effect of KPL-4 transplanted tumours. The values represent mean tumour volumes of ten tumours (**A**) or mean bodyweights of five mice (**B**) in each group (○, control; •, MPA-treated; △, intact mice). Bars, s.e. $^*P < 0.05$; $^{**}P < 0.01$ compared with respective controls complete recovery of bodyweight loss in the mice bearing KPL-4 tumours.

The serum IL-6 levels of the recipient mice were also measured 7 weeks after the cell injections. The values were 57.6 ± 20.3 pg ml$^{-1}$ for the control group and 8.9 ± 2.5 pg ml$^{-1}$ for the MPA-treated group. MPA significantly reduced serum IL-6 levels in nude mice bearing KPL-4 tumours ($P$ = 0.019). IL-1$\beta$, IL-11, TNF-$\alpha$, leukaemia inhibitory factor and γ-interferon were not detectable in the serum of the mice bearing KPL-4 tumours in a preliminary experiment. In addition, the IL-6 concentration in the MPA-treated tumours was significantly lower than that in the control tumours (data not shown). Histological examination revealed no remarkable change in tumour cell morphology or mononuclear cell infiltration in the MPA-treated tumours.

### DISCUSSION

MPA is a synthetic analogue of progesterone, which has been widely used in clinics as a second-line endocrine therapeutic agent for the treatment of patients with recurrent breast cancer. Although the efficacy of MPA has been reported to be comparable or superior to that of tamoxifen, a non-steroidal anti-oestrogen, tamoxifen has been recognized as a first-line endocrine therapeutic agent because of fewer adverse effects (Van Veelen et al, 1986). One of the main adverse effects of MPA is weight gain of patients. However, the results of a recent clinical trial suggest that this adverse effect results in improvement of the anorexia and bodyweight loss induced in patients by cytotoxic agents (Tominaga et al, 1994). Moreover, MPA has been used as an anticachectic agent for patients with non-breast malignancies in a clinical trial (Downer et al, 1993). A similar clinical trial has been conducted using another synthetic progestin, megestrol acetate (Gebbia et al, 1996). To explore the action mechanisms of MPA responsible for the anticachectic effect, some clinical and experimental studies have been conducted. A very recent report suggested that MPA reduces the production of cytokines including IL-6 by peripheral blood mononuclear cells of cancer patients (Montovani et al, 1997). Another report suggested that MPA reduces the serum IL-6 levels of patients with advanced breast cancer and preserves the bodyweight of these patients (Yamashita et al, 1996). Although a series of experimental studies and some clinical studies suggest that IL-6 secreted from tumour cells may mediate cancer-induced cachexia, the effects of MPA on IL-6 production and secretion in tumour cells still remain to be elucidated. These findings prompted us to explore the direct action of MPA on IL-6 expression from human breast cancer cells.

We recently established a new human breast cancer cell line, KPL-4, from a patient with recurrent breast cancer (Kurebayashi et al, 1997). During an animal experiment, we unexpectedly found that KPL-4 transplanted tumours induced severe cachexia in the recipient mice. Subsequent studies revealed that KPL-4 cells secrete immunoreactive IL-6 into medium and that serum IL-6 levels correlate with the weights of KPL-4 tumours in recipient mice (Kurebayashi et al, 1998). These findings suggest that IL-6 secretion from KPL-4 cells may be a main cause of cachexia in this model system. Therefore, in this study, we decided to use this

© Cancer Research Campaign 1999

model system to explore possible mechanisms of action of MPA responsible for cancer-induced cachexia.

In the present study, we demonstrate for the first time that MPA significantly reduces both IL-6 secretion into medium and IL-6 mRNA levels in a human breast cancer cell line (Figure 1). Moreover, MPA also suppresses TNF-α-stimulated IL-6 secretion and IL-6 mRNA levels in this cell line (Figure 2). TNF-$\alpha$ is known to be one of the most potent activators of IL-6 expression (Kishimoto, 1989). Because we supposed that IL-6 secretion from tumour cells should be regulated by some other cytokines through paracrine pathways in vivo, we chose to investigate TNF-$\alpha$ in this study. In addition, the serum IL-6 levels of mice bearing breast tumours were lowered by MPA without affecting tumour growth (Figure 3A). This result agrees with clinical data showing that a reduction in serum IL-6 levels of patients with advanced breast cancer do not correlate with the anti-tumour effect of MPA (Yamashita et al, 1996). More interestingly, the reduction in the serum IL-6 levels induced by MPA correlated with reversion of bodyweight loss in the recipient mice (Figure 3B). These findings suggest that a direct effect of MPA on IL-6 secretion from tumour cells may be the main cause of the anticachectic effect of MPA. In our preliminary experiment, IL-1$\beta$, IL-11, TNF-$\alpha$, leukaemia inhibitory factor and $\gamma$-interferon were not detectable in the serum of the mice bearing KPL-4 tumours. However, because some other cachectic factors have been reported and the serum levels of these factors were not systematically investigated in this study, it might be possible that another factor is the main cause of cachexia in this model system. Therefore, further analysis is needed to clarify the role of IL-6 in cancer-induced cachexia.

IL-6 production and secretion are known to be stimulated by lipopolysaccharide and cytokines, such as TNF-$\alpha$ and IL-1 (Okamoto et al, 1996*a,b*) in some tumour cells. The regulatory mechanisms of IL-6 expression have been also studied in various normal cells, such as fibroblasts, macrophages, endothelial cells, epidermal cells, keratinocytes and synovial cells (Kishimoto, 1989). However, only in a recent report has it been suggested that MPA reduces IL-6 secretion from the peripheral blood mononuclear cells of cancer patients (Montovani et al, 1997). MPA is known to bind not only to progesterone receptors but also to glucocorticoid and androgen receptors (Poulin et al, 1989; Narita et al, 1995) Because KPL-4 cells used in this study do not express either oestrogen or progesterone receptors, it is conceivable that MPA may act as a glucocorticoid or androgen through glucocorticoid or androgen receptors. Our preliminary results suggest that hydrocortisone also reduces both basal and TNF-α-stimulated IL-6 secretion from KPL-4 cells (unpublished data). Further analysis is needed to elucidate the signalling pathway of MPA responsible for the inhibition of IL-6 expression in this cell line.

### ACKNOWLEDGEMENTS

This work was supported in part by a grant from the Ministry of Education, Science, Sports and Culture of Japan and by Research Project Grants (no. 8-301, 9-106) from Kawasaki Medical School.

### REFERENCES

Beutler B and Cerani A (1986) Cachectin and tumor necrosis factor as two sides of the same biological coin. *Nature* **320**: 584–588

Billingsley KG, Fraker DL, Strassmann G, Loeser C, Fliot HM and Alexander HR (1996) Macrophage-derived tumor necrosis factor and tumor-derived leukemia inhibitory factor and interleukin-6: possible cellular mechanisms of cancer cachexia. *Ann Surg Oncol* **3**: 29–35

Chiu JJ, Sgagias MK and Cowan KH (1996) Interleukin 6 acts as a paracrine growth factor in human mammary carcinoma cell lines. *Clin Cancer Res* **2**: 215–221

De La Mata J, Uy HL, Guise TA, Story B, Boyce BF, Mundy GR and Roodman GD (1995) Interleukin-6 enhances hypercalcemia and bone resorption mediated by parathyroid hormone-related protein in vivo. *J Clin Invest* **95**: 2846–2852

Downer S, Joel S, Allbright A, Plant H, Stubbs L, Talbot D and Slevin M (1993) A double blind placebo controlled trial of medroxyprogesterone acetate (MPA) in cancer cachexia. *Br J Cancer* **67**: 1102–1105

Fujimoto-Ouchi K, Tamura S, Mori K, Tanaka Y and Ishistuka H (1995) Establishment and characterization of cancer-inducing and non-inducing clones of murine colon 26 carcinoma. *Int J Cancer* **61**: 522–528

Gebbia V, Testa A and Gebbia N (1996) Prospective randomized trial of two doses of megestrol acetate in the management of anorexia–cachexia syndrome in patients with metastatic cancer. *Br J Cancer* **73**: 1576–1580

Greenberg SA, Nordan RP, McIntosh J, Calvo JC, Scow RO and Jablons D (1992) Interleukin 6 reduces lipoprotein lipase activity in adipose tissue of mice in vivo and in 3T3-L1 adipocytes: a possible role for interleukin 6 in cancer cachexia. *Cancer Res* **52**: 4113–4116

Kajimura N, Iseki H, Tanaka R, Ohue C, Otsubo K, Gyoutoku M, Sasaki K, Akiyama Y and Yamaguchi K (1996) Toxohormones responsible for cancer cachexia syndrome in nude mice bearing human cancer cell lines. *Cancer Chemother Pharmacol* **38**: 48–52

Kawano M, Hirano T, Matsuda T, Taga T, Horii Y, Iwato K, Asaoka H, Tang B, Tanabe O, Tanaka H, Kuramoto A and Kishimoto T (1988) Autocrine generation and requirement of BSF-2/IL-6 for human multiple myelomas. *Nature* **332**: 83–85

Kishimoto T (1989) The biology of interleukin 6. *Blood* **74**: 1–10

Kurebayashi J, Tang CK, Kurosumi M and Sonoo H (1997) Establishment of a new human breast cancer cell line, KPL-4, overexpressing three members of the Erb B family. *Proc Am Assoc Cancer Res* **38**: 236

Kurebayashi J, Tang CK, Otsuki T, Kurosumi M, Yamamoto S, Tanaka K, Mochizuki M, Nakamura H and Sonoo H (1999) Isolation and characterization of new human breast cancer cell line, KPL-4, expressing Erb B family receptors and interleukin-6. *Br J Cancer* (in press)

Matthys P, Dijkmans R, Proost P, Van Damme J, Hermans H, Sobis H and Billiau A (1991) Severe cachexia in mice inoculated with interferon-γ-producing tumor cells. *Int J Cancer* **49**: 77–82

Metcalf D, Nicola NA and Gearing DP (1990) Effects of leukemia inhibitory factor on hematopoietic and other tissues in mice. *Blood* **76**: 50–56

Miki S, Iwano M, Miki Y, Yamamoto M, Tang B, Yokokaya K, Sonoda T, Hirano T and Kishimoto T (1989) Interleukin-6 (IL-6) functions as an in vitro autocrine growth factor in renal cell carcinomas. *FEBS Lett* **250**: 607–610

Moldawer LL, Andersson C, Glein J and Lundholm KG (1988) Regulation of food intake and hepatic protein synthesis by recombinant-derived cytokines. *Am J Physiol* **254**: 450–456

Montovani G, Maccio A, Esu S, Lai P, Santona MC, Massa E, Dessi D, Melis GB and Del Giacco GS (1997) Medroxyprogesterone acetate reduces the in vitro production of cytokines and serotonin involved in anorexia/cachexia and emesis by peripheral blood mononuclear cells of cancer patients. *Eur J Cancer* **33**: 602–607

Mori K, Fujimoto-Ouchi K, Ishikawa T, Sekiguchi F, Ishitsuka H and Tanaka Y (1996) Murine interleukin-12 prevents the development of cancer cachexia in a murine model. *Int J Cancer* **67**: 849–855

Narita T, Kawakami-Kimura N, Matsuura N, Hosono J and Kannagi R (1995) Corticosteroids and medroxyprogesterone acetate inhibit the induction of E-selectin on the vascular endothelium by MDA-MB-231 breast cancer cells. *Anticancer Res* **15**: 2523–2527

Ohe Y, Podack ER, Olsen KJ, Miyahara Y, Miura K, Saito H, Koishihara Y, Ohsugi Y, Oihara T, Nishio K and Saijo N (1993) Interleukin-6 cDNA transfected Lewis lung carcinoma cells show unaltered net tumour growth rate but cause weight loss and shorten survival in syngenic mice. *Br J Cancer* **67**: 939–944

Ohira T, Nishio K, Ohe Y, Arioka H, Nishio M, Funakawa Y, Ogasawara H, Fukuda M, Yazawa K, Kato H and Saijo N (1996) Improvement by eicosanoids in cancer cachexia induced by LCC-IL6 transplantation. *J Cancer Res Clin Oncol* **122**: 711–715

Ohsumi J, Miyadai K, Kawasahima I, Sakakibara S, Yamaguchi J and Itoh Y (1994) Regulation of lipoprotein lipase synthesis in 3T3-L1 adipocytes by interleukin-11/adipogenesis inhibitory factor. *Biochem Mol Biol Int* **32**: 705–712

Oka M, Yamamoto K, Takahashi M, Hakozaki M, Abe T, Iizuka N, Hazama S, Hirazawa K, Hayashi H, Tangoku A, Hirose K, Ishihara T and Suzuki T (1996)

© Cancer Research Campaign 1999 

Relationship between serum levels of interleukin 6, various disease parameters and malnutrition in patients with esophageal squamous cell carcinoma. *Cancer Res* **56**: 2776–2780

Okamoto M, Hattori K and Oyasu R (1997*a*) Interleukin-6 functions as an autocrine growth factor in human bladder carcinoma cell lines *in vitro*. *Int J Cancer* **72**: 149–154

Okamoto M, Lee C and Oyasu R (1997*b*) Interleukin-6 as a paracrine and autocrine growth factor in human prostatic carcinoma cells in vitro. *Cancer Res* **57**: 141–146

Poulin R, Baker D, Poirier D and Labrie F (1989) Androgen and glucocorticoid receptor-mediated inhibition of cell proliferation by medroxyprogesterone acetate in ZR-75-1 human breast cancer cells. *Breast Cancer Res Treat* **13**: 161–172

Strassmann G, Fong M, Kenney JS and Jacob CO (1992*a*) Evidence for the involvement of interleukin 6 in experimental cancer cachexia. *J Clin Invest* **89**: 1681–1684

Strassmann G, Jacob CO, Evans R, Beall D and Fong M (1992*b*) Mechanisms of experimental cancer cachexia. Interaction between mononuclear phagocytes and colon-26 carcinoma and its relevance to IL-6-mediated cancer cachexia. *J Immunol* **148**: 3674–3678

Takemura M, Kiyoshima M, Saito K, Noma A, Shinoda J, Sato M and Kasai C (1996) Evaluation of interleukin-6 (IL-6) measurement by highly sensitive chemiluminescent enzyme immunoassay (Japanese). *Igaku-to-Yakugaku* **36**: 1071–1076

Todorov P, Cariuk P, McDevitt T, Coles B, Fearon K and Tisdale M (1996) Characterization of a cancer cachectic factor. *Nature* **379**: 739–742

Tominaga T, Abe O, Ohshima A, Hayasaka H, Uchino J, Abe R, Enomoto K, Izuo M, Watanabe H, Takatani O, Yoshida M, Sakai K, Koyama H, Hattori T, Senoo T, Monden Y and Nomura Y (1994) Comparison of chemotherapy with or without medroxyprogesterone acetate for advanced or recurrent breast cancer. *Eur J Cancer* **30**: 959–964

Tsujinaka T, Fujita J, Ebisui C, Yano M, Kominami E, Suzuki K, Tanaka K, Katsume A, Ohsugi Y, Shiozaki H and Monden M (1996) Interleukin 6 receptor antibody inhibits muscle atrophy and modulates proteolytic systems in interleukin 6 transgenic mice. *J Clin Invest* **97**: 244–249

Van Veelen H, Willemse PH, Tjabbes T and Sleijfer DT (1986) Oral high-dose medroxyprogesterone acetate versus tamoxifen. A randomized crossover trial in postmenopausal patients with advanced breast cancer. *Cancer* **58**: 7–13

Yamashita J, Hideshima T, Shirakusa T and Ogawa M (1996) Medroxyprogesterone acetate treatment reduces serum interleukin-6 levels in patients with metastatic breast carcinoma. *Cancer* **78**: 2346–2352

Yasumoto K, Mukaida N, Harada A, Kuno K, Akiyama M, Nakashima E, Fujioka N, Mai M, Kasahara T, Fujimoto-Ouchi K, Mori K, Tanaka Y and Matsushima K (1995) Molecular analysis of the cytokine network involved in cachexia in colon 26 adenocarcinoma-bearing mice. *Cancer Res* **55**: 921–927

 © Cancer Research Campaign 1999
